# Empirical evidence of recall bias for primary health care visits

**DOI:** 10.1186/s12913-015-1039-1

**Published:** 2015-09-15

**Authors:** Natasha Kareem Brusco, Jennifer J. Watts

**Affiliations:** Physiotherapy Department, Faculty of Health Science, La Trobe University, Bundoora Campus, Melbourne, VIC 3086 Australia; Physiotherapy Services, Cabrini Health, 183 Wattletree Road, Malvern, VIC 3144 Australia; Allied Health Clinical Research Office, Eastern Health, Level 2, 5 Arnold Street, Box Hill, VIC 3128 Australia; Deakin Health Economics, Population Health Strategic Research Centre, Deakin University, 221 Burwood Highway, Burwood, VIC 3125 Australia

## Abstract

**Background:**

While it is common for an economic evaluation of health care to rely on trial participants for self-reported health service utilisation, there is variability in the accuracy of this data due to potential recall bias. The aim of this study was to quantify the level of recall bias in self-reported primary health care general practitioner (GP) visits following inpatient rehabilitation over a 12 month period.

**Methods:**

This report is a secondary analysis from a larger randomised control trial of an economic evaluation of additional Saturday inpatient rehabilitation. Participants were adults who had been discharged into the community following admission to an acute general rehabilitation hospital. Participants were asked to recall primary health care visits, including community GP visits, via a telephone questionnaire which was administered at 6 and 12 months following discharge from inpatient rehabilitation. Participants were asked to recall health service utilisation over each preceding 6 month period. The self-reported data were compared to equivalent claims data from the national insurer, over the same period.

**Results:**

751 participants (75 % of the full trial) with a mean age of 74 years (SD 13) were included in this analysis. Over the 12 month period following discharge from rehabilitation there was an under-reporting of 14 % in self-reported health service utilisation for GP visits compared to national insurer claims data over the same period. From 0 to 6 months following discharge from rehabilitation, there was an over-reporting of self-reported GP visits of 35 % and from 7 to 12 months there was an under-reporting of self-reported GP visits of 36 %, compared to national insurer claims data over the same period. 46 % of patients reported the same or one number difference in self-reported GP visits between the 0 to 6 and the 7 to 12 month periods.

**Conclusion:**

Based on these findings we recommend that an economic evaluation alongside a clinical trial for an elderly adult rehabilitation population include a sensitivity analysis that inflates self-reported GP visits by 16 % over 12 months. However caution is required when utilising self-reported GP visits as the data may contain periods of both over and under reporting. Where general practitioner visits are expected to vary significantly between intervention and control groups we recommend that administrative data be included in the trial to accurately capture resources for an economic evaluation.

## Background

It is common for an economic evaluation of health care to rely on trial participants for self-reported health service utilisation [1, 2]. Often self-reported health care visits are collected by a questionnaire designed specifically for the individual study to capture the health care resource of interest and this can be administered face to face, by mail out or over the telephone. However, with any self-reported data there is risk of recall bias. Recall bias arises when there is error in the recall of information that may include forgetting an event or recalling an event that did not occur [3] and the reason for this can be multi-factorial [4]. An event may also be recalled with an incorrect timeline, that is, recalling an event prior to the specified period that actually occurred within the specified period (backward telescoping) or recalling an event inside the specified period that actually occurred prior to the specified period (forward telescoping) [4]. In the context of health service utilisation, poor memory may substantially contribute to recall bias with an inability to recall when, and the precise number of times, an event took place [5]. Accuracy of health service self-reported resource utilisation can be influenced by different demographic and methodological factors such as patient population, recall time frame, questionnaire design and mode of data collection [6].

This study involved follow up for 12 months of 996 adults post-discharge from an acute adult rehabilitation facility. The trial intervention was an additional inpatient rehabilitation service on a Saturday. The primary aim of the study was to determine the effect of the additional Saturday service with respect to clinical outcomes and costs [7]. A secondary aim of the research and the aim of the current study, was to quantify the level of recall bias in self-reported primary health care visits, specifically community medical general practitioner (GP) visits, by comparing primary health care visits from a the self-reported questionnaire to those recorded by the national insurer over a 12 month period.

## Methods

This study was a sub-group analysis from an economic evaluation alongside a randomised controlled trial that compared usual care Monday to Friday rehabilitation to Monday to Saturday rehabilitation for a general mixed adult population across two Australian inpatient rehabilitation facilities. The rehabilitation facilities serviced a general mixed adult population and patients were typically admitted to rehabilitation following an acute hospital admission. Diagnoses included routine orthopaedic lower limb joint replacement, falls and fracture management, neurological diagnosis, deconditioning, lower limb amputation, as well as other rehabilitation conditions. Many of the patients admitted were older adults, although age ranged from 18 to 100+ and the average admission was three weeks [8]. Full details of the protocol [9], the clinical outcomes [10] and economic evaluations of the clinical trial [7, 11] have been published elsewhere. The trial was registered with the Australian and New Zealand Clinical Trials Registry (ACTRN12609000973213). The trial was approved by the Eastern Health Research and Ethics Committee (E58 09/10) and La Trobe University Human Research Ethics Committee (FHEC10/14). All patients provided written informed consent for the clinical trial and follow up data collection period, as well as a separate consent to meet requirements for Medicare Australia for Medicare Benefits Schedule and Pharmaceutical Benefit Scheme claims data.

To investigate if there was any recall bias associated with self-reported health service utilisation we compared community general practitioner (GP) claims recorded by the national insurer, Medicare Australia, to self-reported data over the same 12 month period. GP claims were the only variable that was collected by both Medicare and included on the health service utilisation questionnaire. A sub-group of participants was selected from the 996 participants in the full clinical trial and these were the participants who were alive at 12 months, had completed both the 6 and 12 month questionnaires and for whom we had 12 months of national insurer data. A copy of the self-reported questionnaire is available as an Additional File from the publication of the economic evaluation of the inpatient rehabilitation admission including the 12 month follow up period [7].

During the 12 months following discharge from rehabilitation both administrative and self-reported data were collected. Administrative data were collected at 12 months following discharge from rehabilitation and these included data from the primary rehabilitation health service, as well as from the national insurer, Medicare Australia. Data from the primary rehabilitation service included hospital admissions and outpatient services and Medicare data included pharmaceuticals, medical specialist and community GP claims. A minimum six month lag was employed between the end point of data collection to the time of data extraction. This allowed for the Medicare claim data to be processed.

In addition, a health service utilisation questionnaire was administered by telephone at 6 and 12 months following discharge from rehabilitation to supplement the administrative data. The health service utilisation questionnaire was administered by assessors blinded to group allocation in the wider clinical trial. Participants were asked to recall health service utilisation over the preceding 6 month period. Participants reported admissions and length of stay of acute or rehabilitation hospital admissions in facilities outside of the primary rehabilitation health service, community GP visits, non-Medicare health care visits, over-the-counter medications, and the assistance of carers. Assessors completing the follow up telephone calls prompted the participant to include both GP visits in the GP’s consulting rooms as well as GP visits where the GP travelled to the participant’s home environment. Home was defined as place of residence, for example this could include the family home or a residential care facility.

Mean difference between self-reported data and Medicare data were determined using a paired samples *t*-test. Correlation was reported using the Spearman coefficient. The Kappa coefficient (*ĸ*) was calculated to measure beyond chance agreement among measurements of GP visits between self-reported and Medicare data, as well as between the 6 and 12 month data. The self-reported data were also examined to determine the degree of similarity for reporting the same number of GP visits in the two time periods. Likewise, Medicare data were examined to determine the degree of similarity for GP utilisation in the two time periods. All data sets were checked for assumptions of normality using Kolmogorov-Smirnov. IBM SPSS Statistics Version 21 was used for the analyses and to produce the figures [12]. Data were analysed and compared over the full 12 months and for each 6 month period.

## Results

A total of 751 participants (75 %) from the clinical trial had complete data at 12 months and were included in this analysis. Mean age of participants in the recall bias analysis sample was 74 years (standard deviation (SD) 13), 244 (33 %) were men and the sample included 464 (62 %) with an orthopaedic diagnosis, 145 (19 %) with a neurological diagnosis and 142 (19 %) with other disabling conditions. Characteristics for participants from the randomised controlled trial were compared between those who were and were not included in the recall bias analysis (Table 1). People included in the sample were more likely to be younger, female, have an orthopaedic diagnosis and a shorter length of stay in rehabilitation. There were also significant differences with people in the recall sample having less comorbidities, more likely to be living independently in the community prior to admission and a higher functional status on admission and discharge from rehabilitation. In addition, participants who were included in the recall bias sample received more therapy per week during the rehabilitation admission and had a reduced length of stay, although they were not more likely to be in the intervention group.

**Table 1:** Characteristics of patients from the randomised controlled trial who were and were not included in the recall bias analyses

	Patients who were included in the recall bias sample (*n* = 751)	Patients who were not included in the recall bias sample (*n* = 245)
Age, mean (SD)	73.8 (12.5)	76.4 (13.4)*
Gender, n male (%)	224 (33)	115 (47)*
Diagnosis category, n orthopaedic (%)	464 (62)	102 (42)*
Charlson comorbidity index, mean (SD)	0.91 (1.3)	1.38 (1.6)*
Intervention group of the randomized controlled trial, n (%)	371 (49)	125 (51)
Rehabilitation length of stay, mean days (SD)	21.1 (16.9)	25.4 (21.0)*
Therapy per week during rehabilitation admission, mean (SD)	488.3 (171.6)	438.8 (181.4)*
Functional independence (FIM) on admission, mean (SD)	86.2 (17.5)	76.6 (23.1)*
Functional independence (FIM) on discharge, mean (SD)	107.9 (14.3)	94.6 (27.5)*
Health-related quality of life EQ-5D utility index on admission, mean (SD)	0.34 (0.35) (*n* = 721)	0.37 (0.36) (*n* = 222)
Health-related quality of life EQ-5D utility index on discharge, mean (SD)	0.65 (0.26) (*n* = 699)	0.63 (0.29) (*n* = 205)
Living independently in the community prior to admission, n (%)	713 (97) (*n* = 737)	218 (93) (*n* = 235)*
Living independently in the community following discharge, n (%)	616 (86) (*n* = 720)	200 (92) (*n* = 221)

* = *p* < 0.05

There were 36 different Medicare item numbers pertaining to a GP community visit that were included in this analysis, from the Medicare administrative data set. The three most common Medicare item categories were Consultation at Consulting Rooms – Level B (14 %); Initiation of a Patient Episode (8 %); and Prothrombin time (including INR) (7 %).

**Fig. 1:**
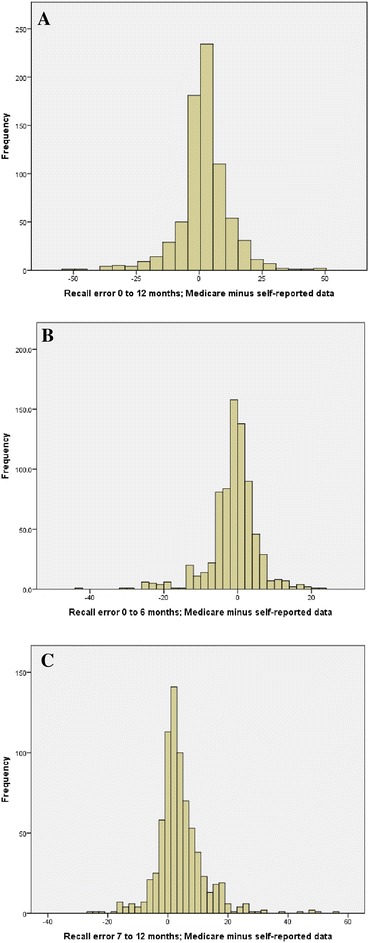
Recall error between the Medicare data and the self-reported data for the different time periods **a**. Recall error 0 to 12 months. **b**. Recall error 0 to 6 months. **c**. Recall error 7 to 12 months

The mean number of self-reported community GP visits per person over 12 months was 12.5 (SD 10.3) (median 10) with a total of 9393 visits. The mean number of Medicare recorded community GP claims was 14.5 (SD 10.3) (median 13) with a total of 10,855 visits (Table 2). There was a mean difference of 1.9 visits (95 % CI 1.2 to 2.7, *p* < 0.01), that represented a significant under-reporting of self-reported community GP visits with very low levels of correlation or agreement (Table 3 and Fig. 1a). Using administrative data from the national insurer and comparing this to the self-reported data for community GP visits over 12 months, an under-reporting of 13.5 % was identified for self-reported data, or expressed another way, self-reported data would need to be inflated by 15.6 % to equate to the data from the national insurer. Data presented in Table 2 on the number of GP visits did not violate assumptions of normality (Kolmogorov-Smirnov- test, *p* > 0.05).

**Table 2:** Medicare general practitioner claims (national insurer) compared to self-reported general practitioner visits in the twelve months following discharge from rehabilitation (*n* = 751)

	0-6 Months			7-12 Months			0-12 Months (combined)		
	Medicare GP claims	Self-reported GP visits	Difference (Medicare – Self-reported)	Medicare GP claims	Self-reported GP visits	Difference (Medicare – Self-reported)	Medicare GP claims	Self-reported GP visits	Difference (Medicare – Self-reported)
Number of GP visits	3,441	4,653	−1,212	7,414	4,740	2,674	10,855	9,393	1,462
Median (range)	3	5	−1*	8	5	2*	13	10	2*
	(0 to 29)	(0 to 52)	(−43 to 22)	(0 to 65)	(0 to 70)	(−26 to 56)	(0 to 76)	(0 to 100)	(−52 to 50)
Mean (SD)	4.6	6.2	−1.6	9.9	6.3	3.6	14.5	12.5	1.9
	(5.0)	(5.8)	(CI −2.1 to −1.1)	(8.7)	(6.2)	(CI 3.0 to 4.1)	(10.3)	(10.3)	(CI 1.2 to 2.7)
			*p* = <0.01			*p* = <0.01			*p* = <0.01

*GP* general practitioner

*Median of the difference

**Table 3:** Correlation of Medicare general practitioner claims (national insurer) and self-reported general practitioner visits (*n* = 751)

	Medicare GP claims compared to self-reported GP visits 0 to 6 months	Medicare GP claims compared to self-reported GP visits 7 to 12 months	Medicare GP claims compared to self-reported GP visits 0 to 12 months	Self-report GP visits 0 to 6 months compared to 7 to 12 months	Medicare GP claims 0 to 6 months compared to 7 to 12 months
Correlation, Spearman	0.25	0.65	0.52	0.53	0.04
	*p* < 0.001	*p* < 0.001	*p* < 0.001	*p* < 0.001	*p* = 0.28
Measure of agreement, Kappa	0.03^a^	0.04^a^	0.03^a^	0.18^a^	0.07^a^
Number of occasions the data matches for the individual participant n (%)	70 (9.3)	67 (8.9)	52 (6.9)	208 (27.7)	91 (12.1)

^a^ = slight agreement (0.0 to 0.2); *GP* general practitioner

From 0 to 6 months following discharge from rehabilitation, there was an over-reporting of self-reported GP visits by 1.6 sessions (95 % CI 2.1 to 1.1; *p* < 0.01) with very low levels of correlation or agreement (Tables 2, 3 and Fig. 1b). This indicates over-reporting of 35.2 % for self-reported data, alternately, self-reported data would need to be deflated by 26.0 % to equate to data from the national insurer. In the second 6 month period from 7 to 12 months following discharge from rehabilitation, there was an under-reporting of self-reported GP visits by 3.6 visits (95 % CI 3.0 to 4.1; *p* < 0.01) with very low levels of correlation or agreement (Table 3 and Fig. 1c). This indicates under-reporting of 36.1 % for self-reported data, alternately, self-reported data would need to be inflated by 56.4 % to equate to data from the national insurer.

**Fig. 2:**
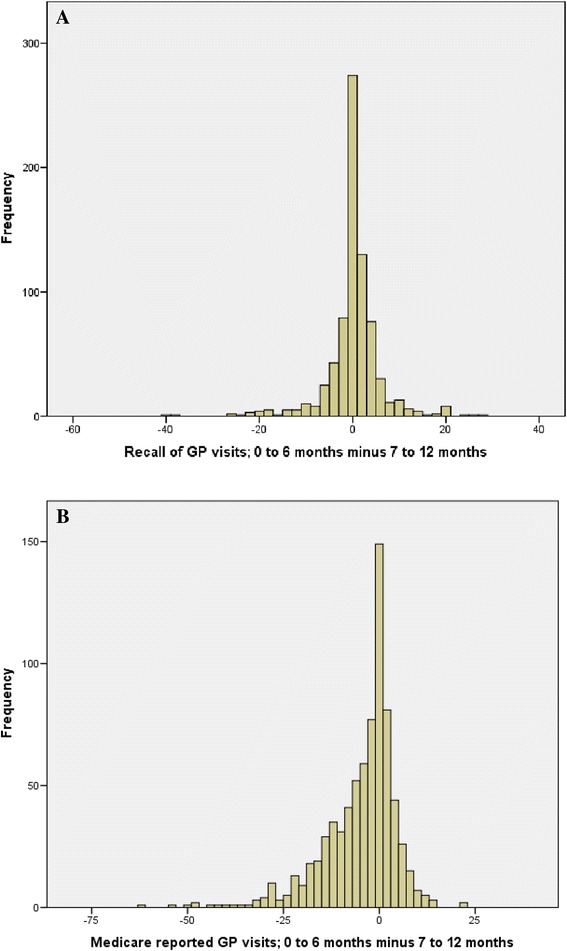
The amount of similarity between data in the two time periods, for the self-reported recall of general practitioner visits and for the Medicare reported general practitioner visits. **a**. Recall of GP visits; 0 to 6 months minus 7 to 12 months. **b**. Medicare reported GP visits; 0 to 6 months minus 7 to 12 months

The number of self-reported GP visits has been compared between the 0 to 6 and the 7 to 12 month period. The results have been presented as a histogram (Fig. 2a). From the health utilisation questionnaire, 208 (28 %) patients reported the same number for both periods, 66 (9 %) reported one less, 73 (10 %) reported one more, 52 (7 %) reported 2 less and 57 (8 %) reported 2 more. This means that 46 % of patients reported the same or one number different between the two periods. In contrast, the Medicare data were also compared between the 0 to 6 and the 7 to 12 month period. The results have been presented as a histogram (Fig. 2b). 91 (12 %) patients had the same number for both periods, 58 (8 %) reported one less, 46 (6 %) reported one more, 40 (5 %) reported 2 less and 35 (6 %) reported 2 more. This means that 26 % of patients had the same or one number different between the two periods based on Medicare data. The correlation and agreement between each of these combinations is presented in Table 3.

## Discussion

Data collection forms for collecting resource utilisation are commonly used in an economic evaluation alongside a randomised controlled trial [1]. Although recall bias is a potential issue in this form of data collection, the impact of recall bias is not known for a mixed adult population following a rehabilitation admission over a 12 month period. Our finding of under-reporting over 12 months is consistent with a literature review that found that under-reporting is more common than over-reporting for recall of primary health care GP visits [6]. This contrasts with recall for hospitalisation where the number of nights in hospital were found to be over-reported by 17 % [1]. In addition to different service types other factors influencing recall bias include the population surveyed, the length of the recall period, the presence of a salient event and in the administration of the questionnaire [6]. We tested the recall of people discharged from an Australian rehabilitation hospital for visits to a community GP, over two recall periods of 6 months in a survey conducted via the telephone.

While overall the recall bias was 13.5 % under-reporting, when the results were broken down into two different time periods (0–6 months and 7–12 months post discharge from rehabilitation), we found mixed results with over-reporting of 35.2 % in the first 6 months following discharge and under-reporting of 36.1 %, in the second 6 month period after discharge. This unexpected result may be influenced by multiple factors and this is further explored, although it is noted that it is not uncommon for data sets to contain both under and over-reporting [6, 2].

For the 0–6 month period it is assumed that the recall of services outside the time point of interest, known as forward telescoping, may have been less of an issue as there was a salient event, the rehabilitation admission. The rehabilitation admission could be used as a memory aid to assist recall to the correct point in time [6]. Yet there was significant over-reporting for this time period. There are two potential explanations. The first is based on the premise that a population, who had just been discharged from rehabilitation, would typically receive a suite of health care services to support the transition from the hospital to the community and to continue rehabilitation. Other types of health care utilisation were high in this group of participants in the recall analysis, with around 17 non-Medicare health care visits self-reported in the same 6 months following discharge from rehabilitation. Recall from 0 to 6 months may have been influenced by an inability to distinguish GP visits from these additional health care services. As such, a possible explanation for over-reporting may be that when participants were asked to recall the different types of visits under separate questions, there may have been inaccuracy when differentiating GP visits from other non-admitted health services in the first 6 months following discharge.

The second explanation is that for these patients, a hospital admission for rehabilitation is not a significant salient event to frame the commencement of the recall period. The participants in the recall analysis had on average 8 days additional hospitalisation in the 6 month period following discharge from rehabilitation. This suggests that using the rehabilitation admission as a memory aid may be a strategy with limitations in this patient population who are exposed to high levels of admitted and non-admitted health care services. It is noted that this is in contrast to previous literature that report a salient event, such as a hospital admission, improves the ability to distinguish events occurring within the recall period and therefore improves accuracy [6].

Recall from the 7 to 12 month period following discharge, which was significantly under-reported, may have been influenced by memory decay (forgetting visits) or forward telescoping (assuming that events are outside of the timeframe) as this is common in recall periods of 6 months or greater [6]. This recall period did not have the salient event of the rehabilitation admission to define the start of the recall period. An explanation is that while there was an increase in the frequency of Medicare reported visits in the period from 7 to 12 months compared to 0–6 months, patients may have recalled a lower average number over the whole 12 months and applied this lower number to the period from 7 to 12 months.

Comparing the results from the first and second recall periods in the recall analysis, it was noted that patients reported a mean of six visits for both periods. From the Medicare data there were a mean of five visits in the 0–6 month period and a mean of 10 visits in the 7–12 month period, a substantial increase in the latter period. However there was a reversed pattern of self-reported utilisation for non-Medicare admitted and non-admitted health care services. In the 7–12 month period there were 10 non-Medicare health care visits compared to 17 non-Medicare health care visits in the 0–6 month period. The inability to differentiate GP services from other health care services may have resulted in patients reporting a mean of six visits for both periods.

Economic analyses alongside clinical trials relying on self-reported data should account for potential recall bias, particularly when self-reported data provides a significant contribution to the final resource utilisation included in an economic evaluation. This can be done via a sensitivity analysis of the included self-reported data that may consider inflating, deflating or removing the self-reported health services. Within trial economic analyses should also consider measuring potential recall bias by comparing a health services that are captured in both administrative and self-reported data. Strengths of this study include a large sample size across two 6 month periods of data collection, providing a combined recall period of 12 months [6], noting that recent literature suggests a total recall period of 12 months may improve accuracy of recall [2].

This study is limited to a mixed adult population that had been discharged from inpatient rehabilitation. Other limitations include multiple assessors and therefore potential variation in the wording or prompting provided to study participants in the recall of health service utilisation. A telephone interview was used to administer the questionnaire which does not allow the same visual cues to prompt recall of information compared to face-to-face interviews. There is potential for bias in the sample of participants included in this sub-group analysis. It has been noted that compared to participants excluded from the analysis, those included were younger, had less comorbidities, and higher functional status on admission and discharge from rehabilitation. Patients in poorer health who are older and have reduced cognition are more likely to under-report [6], therefore the under-reporting in this study may be a conservative estimate for a mixed adult population following a rehabilitation admission. In addition, while Medicare has been shown to have a high correlation of accuracy when compared to the Cancer Registry in Australia [13], it is acknowledged that there are limitations to this data set and there is limited literature reporting the accuracy and external validity of this data [14].

The salient event of the rehabilitation admission is common to all patients at the start of the data collection period. What is varying in the study is whether there is a landmark event to bind the start of the two six month recall periods (0 to 6 months and 7 to 12 months post discharge). It is noted that these results are in contrast to previous literature of how such a memory cue would affect recall. The recall bias examined in this analysis only pertained to community GP visits, not more salient events such as an inpatient admissions that are reported to have a greater degree of recall accuracy [6]. Therefore the results of this study are only generalizable to community health care visits, not hospital admissions. Future consideration may be given to resource data collection forms that further probe as to the nature of the health care visit to improve accuracy.

## Conclusion

Self-reported health service utilisation is a commonly used method for collecting resource use data when conducting an economic evaluation alongside a clinical trial. Our findings give direction and magnitude for recall bias associated with self-reported general practitioner visits compared to Medicare data over 12 months following an acute rehabilitation admission. Based on these findings we recommend that an economic evaluation alongside a clinical trial for an elderly adult rehabilitation population include a sensitivity analysis that inflates self-reported GP visits by 16 % over 12 months. However caution is required when utilising self-reported GP visits as the data may contain periods of both over and under reporting. Where general practitioner visits are expected to vary significantly between intervention and control groups we recommend that administrative data be included in the trial to accurately capture resources for an economic evaluation.

## References

[1] Clarke PM, Fiebig DG, Gerdtham UG (2008). Optimal recall length in survey design. J Health Econ.

[2] Kjellsson G, Clarke P, Gerdtham U-G (2013). Forgetting to remember or remembering to forget: a study of the recall period length in health care survey questions. J Health Econ.

[3] Hassan E (2006). Recall bias can be a threat to retrospective and prospective research designs. Internet J Epidemiol.

[4] Gaskell GD, Wright DB, O’Muircheartaigh CA (2000). Telescoping of landmark events: Implications for survey research. Public Opin Q.

[5] Mistry H, Buxton M, Longworth L (2005). Comparison of general practitioner records and patient self-report questionnaires for estimation of costs. Eur J Health Econ.

[6] Bhandari A, Wagner T (2006). Self-reported utilization of health care services: improving measurement and accuracy. Med Care Res Rev.

[7] Brusco NK, Watts JJ, Shields N, Taylor NF (2015). Is cost effectiveness sustained after weekend inpatient rehabilitation? 12 month follow up from a randomized controlled trial. BMC Health Serv Res.

[8] Brusco NK, Shields N, Taylor NF, Paratz J (2007). A Saturday physiotherapy service may decrease length of stay in patients undergoing rehabilitation in hospital: a randomised controlled trial. Aust J Physiother.

[9] Taylor NF, Brusco NK, Watts JJ, Shields N, Peiris C, Sullivan N, Kennedy G, Teo CK, Farley A, Lockwood K, Radia-George C (2010). A study protocol of a randomised controlled trial incorporating a health economic analysis to investigate if additional allied health services for rehabilitation reduce length of stay without compromising patient outcomes. BMC Health Serv Res.

[10] Peiris C, Shields N, Brusco NK, Watts JJ, Taylor NF (2013). Additional Saturday rehabilitation improves functional independence and quality of life and reduces length of stay: a randomized controlled trial. BMC Med.

[11] Brusco NK, Watts JJ, Shields N, Taylor NF (2014). Are weekend inpatient rehabilitation services value for money? An economic evaluation alongside a randomized controlled trial with a 30 day follow up. BMC Med.

[12] IBM Corp. Released 2012. IBM SPSS Statistics for Windows, Version 21.0. Armonk, NY: IBM Corp.

[13] Goldsbury DE, Smith DP, Armstrong BK, O’Connell DL (2011). Using linked routinely collected health data to describe prostate cancer treatment in New South Wales, Australia: a validation study. BMC Health Serv Res.

[14] Trevena JA, Rogers KD, Jorm LR, Churches T, Armstrong B (2013). Quantifying under-reporting of pathology tests in Medical Benefits Schedule claims data. Aust Health Rev.

